# Role of Sphingosine-1-Phosphate Signaling Pathway in Pancreatic Diseases

**DOI:** 10.3390/ijms252111474

**Published:** 2024-10-25

**Authors:** Fei Fu, Wanmeng Li, Xiaoyin Zheng, Yaling Wu, Dan Du, Chenxia Han

**Affiliations:** 1 West China Center of Excellence for Pancreatitis, Institute of Integrated Traditional Chinese and Western Medicine, West China Hospital, Sichuan University, Chengdu 610041, China; fufei9940@wchscu.cn; 2 Advanced Mass Spectrometry Center, Research Core Facility, Frontiers Science Center for Disease-Related Molecular Network, West China Hospital, Sichuan University, Chengdu 610213, China; liwanmeng@wchscu.cn (W.L.); 2023224070047@stu.scu.edu.cn (X.Z.); 2022224070024@stu.scu.edu.cn (Y.W.)

**Keywords:** S1P, SPHKs, S1PRs, acute pancreatitis, chronic pancreatitis, pancreatic cancer

## Abstract

Sphingosine-1-phosphate (S1P) is a sphingolipid metabolic product produced via the phosphorylation of sphingosine by sphingosine kinases (SPHKs), serving as a powerful modulator of various cellular processes through its interaction with S1P receptors (S1PRs). Currently, this incompletely understood mechanism in pancreatic diseases including pancreatitis and pancreatic cancer, largely limits therapeutic options for these disorders. Recent evidence indicates that S1P significantly contributes to pancreatic diseases by modulating inflammation, promoting pyroptosis in pancreatic acinar cells, regulating the activation of pancreatic stellate cells, and affecting organelle functions in pancreatic cancer cells. Nevertheless, no review has encapsulated these advancements. Thus, this review compiles information about the involvement of S1P signaling in exocrine pancreatic disorders, including acute pancreatitis, chronic pancreatitis, and pancreatic cancer, as well as prospective treatment strategies to target S1P signaling for these conditions. The insights presented here possess the potential to offer valuable guidance for the implementation of therapies targeting S1P signaling in various pancreatic diseases.

## 1. Introduction

The pancreas consists of two primary components: the exocrine pancreas, which constitutes the majority of the pancreatic tissue responsible for secreting digestive enzymes, and the endocrine pancreas, composed of pancreatic islet cells that produce hormones [1,2]. Pancreatic diseases such as pancreatitis and pancreatic cancer (PC) can lead to the primary loss of functional parenchyma and/or the secondary impairment of exocrine pancreatic function [3]. These conditions may result from genetic factors, a high-fat diet, alcohol consumption, the presence of pancreatic duct stones, or other etiological factors [4,5,6].

Pancreatitis is a prevalent exocrine inflammatory condition of the pancreas, which includes both acute pancreatitis (AP) and chronic pancreatitis (CP) [7]. AP is characterized by edema, acinar cell necrosis, hemorrhage, and severe inflammation of the pancreas, typically manifesting as sudden onset and severe abdominal pain [8,9]. Meanwhile, CP is a clinical condition that develops as a result of the ongoing inflammation and chronic fibrosis of the pancreatic acini, leading to irreversible structural damage [10]. PC, often referred to as the “King of Cancer”, is a highly lethal malignancy, resulting in a considerable economic burden on families and society [11]. Notably, recurrent AP can give rise to CP, which stands as a prominent etiological factor in the development of PC [12,13]. Furthermore, the developmental defects of pancreas (DDP) are closely related to these pancreatic disorders [14]. However, the understanding of the pathogenesis underlying these pancreatic diseases remains incomplete, and there is currently a lack of specific treatments available. Recently, increasing evidence has suggested that sphingosine-1-phosphate (S1P)-associated cellular and biological mechanisms participated in the onset and progression of these pancreatic diseases.

Sphingolipids are essential components of all eukaryotic membranes [15]. S1P acts as a bioactive sphingolipid that is involved in various cellular processes including cell proliferation, survival, adhesion, and migration [16]. It is produced through the phosphorylation of sphingolipids by sphingosine kinases (SPHKs). S1P may transmit signals to cells via five specific G protein-coupled receptors (known as S1PR1–5) that are located on the cell membrane [17]. In recent decades, the advancement in the molecular targeting of S1P signaling has become a significant focus of research in autoimmune diseases, inflammatory diseases, tumors, and associated areas [18,19,20,21,22,23,24]. In the case of pancreatic disease, S1P-mediated signaling has been identified as a crucial regulator in pancreatitis [25,26] and PC [27,28], as well as in the early development of the pancreas [29,30]. In this review, we provide a comprehensive overview of the regulatory mechanisms of S1P signaling in AP, CP, PC, and DDP. This study emphasizes the importance of sphingolipid metabolism and elucidates the crucial link between S1P signaling and pathological mechanisms, which may provide valuable alternatives to traditional approaches for the management of pancreatic diseases.

## 2. Search Strategy

A comprehensive literature search was conducted using four electronic databases, namely PubMed, Web of Science, Chinese National Knowledge Infrastructure, and Wanfang data from the study’s inception until 27 September 2024. We used search terms and keywords such as “sphingosine-1-phosphate”, “sphingosine kinase”, “S1P receptor”, “pancreas”, “pancreatitis”, “pancreatic cancer”, and “pancreas development”. Moreover, some articles were found through the systematic tracking of citations in other scholarly publications or by accessing reputable journal websites.

We included studies elucidating the roles of SPHKs, S1P, S1PRs or related metabolites in pancreatic diseases including AP, CP, and PC by integrating evidence from mechanistic studies, animal models, and in vitro observations. Studies were excluded if (1) the articles were neither in English nor Chinese; (2) the full texts were not available; (3) they were duplicate or irrelevant studies. The data were sorted and summarized by different pancreatic diseases.

## 3. Basics of the S1P Signaling Pathway

### 3.1. Synthesis, Degeneration, and Transport of S1P

The de novo synthesis pathway (also known as the anabolic pathway) of S1P occurs in the endoplasmic reticulum (ER), leading to the formation of ceramide, the central component of sphingolipids. This process initiates with serine and palmitoyl-CoA, which are converted to 3-ketodihydrosphingosine through the action of serine palmitoyltransferase. Subsequently, 3-ketodihydrosphingosine is metabolized to dihydrosphingosine by 3-ketodihydrosphingosine reductase. This is followed by the synthesis of dihydroceramide via ceramide synthase, and finally, dihydroceramide desaturase catalyzes the conversion of dihydroceramide to ceramide [31,32]. Ceramide can be metabolized into sphingomyelin and glucosylceramide in the Golgi apparatus [18]. Alternatively, the sphingomyelin pathway (also known as the catabolic pathway) of S1P refers to the sphingomyelin metabolizing to ceramide via sphingomyelinase [22]. After ceramide generation, it is further metabolized to sphingosine by ceramidase, and then SPHKs phosphorylate sphingosine to S1P [31,33], which triggers the functions of multiple cellular signals by binding with S1PRs located on the cell membrane. This process of S1P is called inside-out signaling. Notably, the generation of sphingosine occurs only through the degradation of ceramide.

The degradation of S1P occurs through the catalytic action of three distinct enzymes: S1P phosphatases (SPPs), S1P lyase (S1PL), and lipid phosphate phosphohydrolases (LPPs) [34]. In most cellular environments, S1PL irreversibly cleaves S1P into hexadecenal and ethanolamine phosphate. Additionally, two isoforms of SPP (SPP1 and SPP2) selectively dephosphorylate sphingoid base-1-phosphates, including S1P, leading to the generation of sphingosine [35,36]. The degradation of extracellular S1P is exclusively mediated by a specific group of enzymes known as the LPP isoforms [37].

Cells release S1P into the extracellular environment via various transporters. One of these transporters, known as spinster homolog 2 (SPNS2), is an organic ion transporter that operates independently of adenosine triphosphate (ATP) [38]. Recent studies have elucidated its involvement in developmental processes, the maintenance of organ stability, the regulation of circulating S1P levels, and the modulation of inflammatory responses [39]. Members of the ATP-binding cassette (ABC) family, such as ABCA1 and ABCC1 (MRP1), are also implicated to transport S1P in the extracellular space [40].

### 3.2. Sphingosine Kinases

The regulation of SPHK1 and SPHK2 primarily occurs through phosphorylation, but also involves their interaction with other molecules in a spatial and temporal manner. In addition, although there is some overlap in their functions, they can be distinguished based on variances in their subcellular localization and kinetic properties [33]. SPHK1 is primarily found in the cytosol but can be translocated to the plasma membrane upon stimulation by various cytokines and growth factors [41]. This translocation process is regulated through phosphorylation at serine 225 by extracellular signal-regulated kinase 1/2 (ERK1/2), which enhances the enzyme’s activity [42]. Following translocation, SPHK1 exhibits a propensity for associating with cholesterol and sphingolipid-enriched domains, thereby facilitating cellular proliferation and survival [43]. SPHK2, instead, is found in the plasma membrane, ER, mitochondria, and nucleus. Within the nucleus, SPHK2 has the ability to hinder DNA synthesis, leading to cell cycle arrest [44]. Additionally, it can modify histone deacetylases, which impacts the epigenetic regulation of gene expression [45]. In stressful conditions, SPHK2 localizes in the ER where it promotes ceramide production, facilitating apoptotic cell death induction [46].

### 3.3. S1P Receptors (S1PRs)

**Figure 1:**
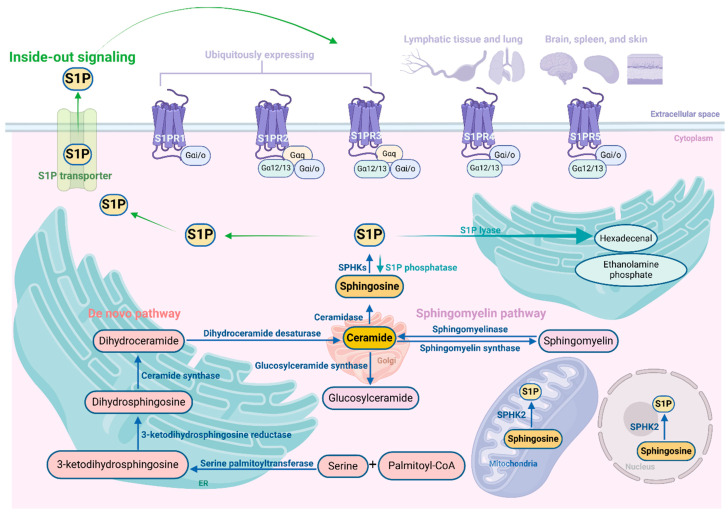
Schematic diagram of S1P signaling pathway.

S1P exerts a paracrine or autocrine function through its binding to S1PRs [47]. In plasma, S1P is mostly produced by erythrocytes, and it binds with chaperones, including apolipoprotein M (ApoM) and albumin in the circulation, and present to S1PRs [22,48]. S1PRs belong to the family of high-affinity G protein-coupled receptors, consisting of five distinct subtypes. S1PRs are selectively coupled to distinct G protein subtypes, with S1PR1 exclusively coupled to heterotrimeric Gαi/o, while S1PR2 and S1PR3 couple to Gαi/o, Gαq, and Gα12/13, and S1PR4 and S1PR5 couple to Gαi/o and Gα12/13 [18,49]. Additionally, S1PRs are widely expressed on numerous cell types and within tissues, yet they exhibit differential expression content in different parts [16]. S1PR1, S1PR2, and S1PR3 are widely present in many tissues, of which S1PR1 is dominant and mainly involved in the regulation of immune trafficking and immune activation [49,50]. While S1PR2 and S1PR3 are mainly involved in the regulation of the endothelial barrier and macrophages, and dendritic cell functions [51]. S1PR4 is mainly expressed in lymphatic and lung tissue, and S1PR5 is mainly expressed in the brain, spleen, and skin [16,52]. Collectively, S1P signaling participates in diverse diseases and drug development, and targeting this system shows promising potential. The schematic diagram of the S1P signaling pathway is shown in Figure 1. The downstream signaling pathways activate multiple cellular signaling such as the Rho family of small GTPases, phosphatidylinositide 3-kinase (PI3K), ERK, and the signal transducer and activator of transcription 3 (STAT3) [18,53]. These pathways subsequently facilitate a multitude of diverse effects in cells, thereby exerting either positive or negative influences on cellular proliferation, survival, and migration [52,54,55]. The dysregulation of the S1P signaling pathway also plays a pivotal role in pancreatic disorders, which is intricately linked to the expression of S1PRs [56,57,58,59].

### 3.4. Current Therapeutic Strategies Targeting S1P Signaling: Regulating SPHKs and S1PRs

Currently, a variety of selective and non-selective modulators targeting SPHKs and S1PRs have been extensively investigated, with some progressing to clinical trials and commercialization as drugs [53,54,60]. Some modulators have been reported to regulate various damage indicators and the proliferation of tumor cells in studies related to pancreatitis and PC [61,62,63], of which FTY720 and JTE-013 were the most studied. Fingolimod (FTY720) is an S1P analog approved by the U.S. FDA as an immunomodulatory drug for treating multiple sclerosis [17,58]. FTY720 can be phosphorylated by SPHK2 to become a biologically active form, FTY720-P, binding to S1PR1-4 and S1PR5 with a particularly high affinity for S1PR1 [64]. Although FTY720-P is an agonist of S1PRs, the binding of FTY720-P to S1PR1 results in the down-modulation and degradation of this receptor, thereby acting as a “functional antagonist” [65]. Moreover, FTY720 can also inhibit the activity of SPHK1 [53]. The representative modulators of S1P signaling are listed in Table 1.

The pancreatic protective effects of FTY720 have been demonstrated in several studies, as evidenced by the attenuation of inflammatory cytokines in AP models [66,67], the inhibition of pancreatic fibrosis in CP rats [68], and the reduction in tumor volume within the pancreas [69]. Similarly, the compound JTE-013 serves as a specific antagonist targeting S1PR2 [70], and is frequently employed to investigate the functional roles of S1PR2 in diverse pancreatic disorders. The administration of JTE-013 significantly ameliorates severe AP-induced intestinal injury [57], while also inhibiting the activation of pancreatic stellate cells and suppressing the proliferation and migration of PC cells [58,71]. In addition, SKI 5c and PF-543 emerge as potent and specific inhibitors of SPHK1 [59,72,73], having also been reported to ameliorate severe AP-induced pulmonary injury [61], and to mitigate pancreatic pathological damage in CP [59], respectively. Several other modulators, such as the dual inhibitors of SPHK1 and SPHK2, N,N-dimethylsphingosine (DMS) and SKI-II, the SPHK2 antagonist opaganib (ABC294640), the S1PR1 and S1PR3 antagonist VPC23019, and the S1PR2 agonist CYM5520, have been extensively utilized in cancer treatment and research [58,74,75,76,77]. Their potential in PC treatment has also been unveiled in recent years. ABC294640, SKI-II, and VPC23019 have been proven to exhibit anti-pancreatic cancer effects [62,78,79]. However, CYM5520 is demonstrated to promote the development of PC [58]. Overall, the modulation of SPHKs and S1PRs with these compounds may provide novel strategies for the management of pancreatic diseases.

**Table 1:** Representative modulators of S1P signaling.

Compound	Structure	Primarily Used As	Effects on Pancreatic Diseases	Ref.
N,N-dimethylsphingosine (DMS)	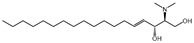	Antagonist of SPHK1 and SPHK2	Ameliorated PC	[74,80,81]
SKI-II	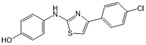	Antagonist of SPHK1 and SPHK2	Ameliorated PC	[62,75,82]
SKI 5c	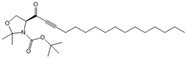	Antagonist of SPHK1	Ameliorated AP	[61,72]
PF-543	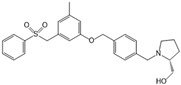	Antagonist of SPHK1	Ameliorated CP	[59,73]
Opaganib (ABC294640)	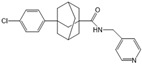	Antagonist of SPHK2	Ameliorated PC	[76,83]
K145	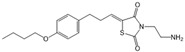	Antagonist of SPHK2	Not available	[84]
Fingolimod (FTY720)	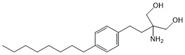	Modulator of S1PR1, S1PR3, S1PR4 and S1PR5, antagonist of SPHK1	Ameliorated AP, CP, and PC	[67,68,69]
Etrasimod (APD334)	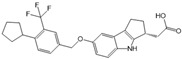	Modulator of S1PR1, S1PR4 and S1PR5	Not available	[85]
Siponimod (BAF312)	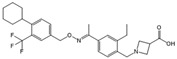	Modulator of S1PR1 and S1PR5	Not available	[86]
Ozanimod (RPC1063)	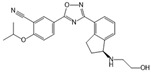	Agonist of S1PR1 and S1PR5	Not available	[87]
VPC23019	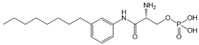	Antagonist of S1PR1 and S1PR3	Ameliorated PC	[77,79]
SEW2871	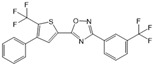	Agonist of S1PR1	AP	[88,89]
Ponesimod (ACT-128800)	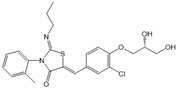	Agonist of S1PR1	Not available	[90]
NIBR-0213	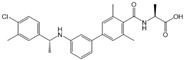	Antagonist of S1PR1	Not available	[91]
CYM5520	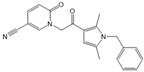	Agonist of S1PR2	Worsened PC	[58]
JTE-013	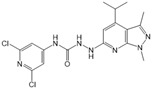	Antagonist of S1PR2	Ameliorated AP, CP, PC	[57,58,70,71]
CYM5541	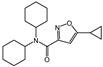	Agonist of S1PR3	Not available	[92]
TY52156	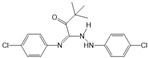	Antagonist of S1PR3	Not available	[91]
CYM50358	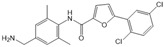	Antagonist of S1PR4	Not available	[93]
A-971432	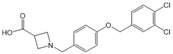	Agonist of S1PR5	Not available	[94]

## 4. Role of S1P Signaling Pathway in Pancreatic Diseases

### 4.1. AP

#### 4.1.1. Functioning as Potential Biomarkers for Severity of AP

Numerous studies have demonstrated that S1P, together with SPHK and S1PRs, functions as significant biomarkers for predicting the severity and prognosis of AP. Clinical evidence suggests that the expression of SPHK1 and S1PR3 in peripheral immune cells such as leukocytes, neutrophils, monocytes, and lymphocytes are significantly elevated in the early stage of AP patients, and then reduce at the restoration stage [95,96,97]. Similarly, a positive correlation between the expression level of SPHK1 and the APACHE II score, a key disease severity evaluating system, are found in AP patients [97]. In accordance with the clinical findings, the S1P concentration and the expression of SPHK1 and S1PR2 in our animal models of AP were elevated, indicating that the activation of S1P signaling may provide a risk factor for the onset of AP [25,26,98]. Our previous study using lipidomic analysis found that in L-ornithine-induced severe AP model rats, in contrast to the decrease in glycerophospholipids, pancreatic ceramide increased significantly, and its metabolic products also increased rapidly at 24 h and peaked at 48 h after modeling, such as glucosylceramides [99]. However, the research regarding the change in S1P levels on AP is inconsistent. As revealed by two clinical studies encompassing 36 severe AP patients and 39 AP patients respectively, the S1P concentration in plasma or serum significantly decreased compared to the control volunteers [100,101]. The different results may be related to the severity and time course of AP, which remains under further investigation. A study revealed plasma S1P concentration significantly increased in mild AP patients on days 1 and 3, accompanied by the inhibition of ceramide synthesis. However, in severe AP patients, S1P decreased, and the level of ceramide increased significantly on days 1 and 3, and they both subsequently returned to normal levels on day 7 [102]. These results suggest that S1P may be a reliable prognostic marker for AP due to its early sensitivity, and shows different changing patterns in mild and severe AP patients.

#### 4.1.2. Mediating Local and Systematic Inflammation

As a severe inflammatory disease, the primary therapeutic measure and objective of AP is to manage inflammation in a targeted manner [103]. S1P signaling is closely associated with inflammatory signal transduction factors, such as IL-6, tumor necrosis factor (TNF)-α, and nuclear factor-kappa B (NF-κB) [104]. It is indicated that the levels of serum proinflammatory cytokines including IL-6, IL-1β, and TNF-α show similar shifts with SPHK1 expression in the peripheral immune cells of severe AP patients, suggesting that the activation of SPHK1 may be closely related to the inflammation of severe AP [97]. Meanwhile, the inhibition of S1PR2 by JTE-013 or genetic knockdown mitigates the severity of pancreatic injury and inflammation levels, as evidenced by a significant reduction in acinar cell death and inflammatory cytokine release, by regulating Rho-associated kinase (ROCK)/NF-κB signaling activation in acinar cells, and macrophage recruitment, as well as polarization towards the M1 phenotype [26]. FTY720, a non-selective S1PR modulator [64,105,106], significantly reduces pancreatic pathological injury and monocyte chemotactic protein-1 (MCP-1) level of hypertriglyceridemic AP mice models [107]. In addition, FTY720 decreases circulative IL-6, IL-10, and TNF-α levels, as well as the number of CD4^+^/CD8^+^ cells in necrotizing AP rats [66]. Although most of the literature indicates that the inhibition of S1P signaling exerts a protective role in AP, similar protective effects have been observed with some S1P signaling agonists [26,61,88,98]. SEW2871, an S1PR1 agonist, significantly ameliorates the parameters of cerulein-induced AP mice, including pancreatic pathological injury and systematic inflammation, which is probably attributed to the inhibition of STAT3 phosphorylation in the pancreas [88].

#### 4.1.3. Inducing Pyroptosis of Pancreatic Acinar Cells (PACs)

PACs are the predominant exocrine cell type undergoing dysfunction in AP [108]. In recent years, there has been a growing interest in mitigating the severity of AP through the intervention targeting of cell pyroptosis in PACs [109,110,111]. Pyroptosis has been observed in the acinar cells of cerulein-induced AP mice through detecting the activation of the pyroptosis characteristic proteins, Caspase-1 and GSDMD. The genetic knockout of *Sphk1* significantly attenuates cell damage and inhibits the pyroptosis of PACs in AP mice [98]. Further in-depth investigation demonstrated that the upregulation of SPHK1 expression in acinar cells is implicated in the regulation of ER stress. The initiation of ER stress triggers protein kinase RNA-like endoplasmic reticulum kinase (PERK) activation, leading to enhanced thioredoxin-interacting protein (TXNIP) expression, which subsequently mediates the Nod-like receptor family and pyrin domain containing 3 (NLRP3) inflammasome activation, and facilitates the generation of active Caspase-1, thereby further promoting cellular pyroptosis [98].

#### 4.1.4. Involvement in AP-Induced Organ Injury

The rapid production and release of a large number of inflammatory cytokines in sever AP can easily lead to systemic inflammatory response syndrome and multiple organ dysfunction syndromes [112]. AP-associated acute lung injury represents the most common and earliest organ dysfunction in disease development, which is characterized by significant pulmonary edema, hyperemia, and inflammatory infiltration in the alveoli [113]. The inhibition of SPHK1 by SKI 5c results in the increased survival rate of AP-associated acute lung injury rats, while concurrently reducing the MPO level in the lung and the protein content of bronchoalveolar lavage fluid [61]. By contrast, others found that S1P signaling shows anti-inflammatory effects and protection against pulmonary injury in AP-associated acute lung injury [67,114]. The intraperitoneal administration of S1P mitigates pulmonary pathological injury, leading to a reduction in inflammatory mediators including IL-1β, IL-6, and TNF-α in bronchoalveolar lavage fluid. Additionally, it inhibits lung immune cell infiltration and suppresses NF-κB activity in alveolar macrophages [67,114]. These conflicting results reflect both the proinflammatory and anti-inflammatory effects of S1P signaling during AP-associated acute lung injury. As a powerful angiogenic factor that strengthens lung endothelial cell integrity and suppresses vascular permeability and alveolar flooding in preclinical animal models of acute lung injury, S1P holds therapeutic potential [115]. It is supposed that the role of S1P signaling in lung inflammation and injury might depend on the type of insult and the degree of oxidative stress, which should be further investigated in long-term studies.

Intestinal damage is another severe complication associated with severe AP, which includes gut mucosal barrier functional changes, intestinal immune barrier dysfunction, and gastrointestinal microbial imbalance, and exaggerates the severity of disease by increasing intestinal permeability, microbial infections, bacterial translocation, and the release of proinflammatory substances [112,116]. The inhibition of S1PR2 by JTE-013 significantly attenuates pathological injury and pyroptosis in the intestinal tissues of severe AP mice [57]. Additionally, S1PR2 was reported to exert a positive regulatory effect on macrophage pyroptosis, and this process might be attributed to the upregulation of the Ras homolog family member A (RhoA)/ROCK signaling pathway [57].

### 4.2. CP

The irreversible fibrosis of the pancreas is the main characteristic of CP [117]. Pancreatic fibrosis is essentially the increase in the synthesis and the decrease in the degradation of the collagen-based extracellular matrix, of which pancreatic stellate cells (PSCs) are key cells involved in pancreatic injury [118]. It has been demonstrated that S1P signaling is closely associated with the development of diverse tissue fibrosis by interacting with excessive extracellular matrix synthesis and high profibrotic marker gene expression [119,120,121,122]. The concentration of S1P has been found to be significantly elevated in in vivo and in vitro CP models [59,71]. It has also been found that the administration of S1P results in the severe impairment of pancreatic function and histomorphology in CP rats via S1P-S1PR2 signaling-mediated autophagy and the NLRP3 inflammasome activation in PSCs [71]. Through further crosstalk experiments, it was revealed that the S1P derived from PACs contributes to the fibrosis of CP by inducing autophagy and activating PSCs through the adenosine monophosphate-activated protein kinase (AMPK)/mammalian target of rapamycin (mTOR) signaling pathway [59]. Moreover, the S1PR modulator FTY720 has been shown to exhibit an anti-fibrotic role by significantly reducing the expression of fibrosis-related regulatory factors, including IFN-γ and transforming growth factor (TGF)-β1 [68]. Due to the non-selectivity of FTY720 in regulating S1PR, how FTY720 regulates S1PR in specific disease models remains elusive, and there is still a lack of in-depth explanation of FTY720 in regulatory mechanisms of pancreatitis. Moreover, since there are a limited number of studies investigating the correlation between S1P signaling and CP, further elucidation is required regarding the corresponding downstream molecular regulatory mechanisms and signaling pathways.

### 4.3. PC

#### 4.3.1. Targeting S1P Signaling for PC Therapy

S1P plays a pivotal role in multiple features of cancer, including the sustained activation of cell proliferation signaling pathways, the evasion of growth inhibitory signals, the resistance to programmed cell death, the promotion of uncontrolled replication, the induction of angiogenesis, and the initiation of invasion and metastasis processes [123,124,125]. Targeting SPHK2 has proven to successfully improve tumor outcomes via PhotoImmunoNanoTherapy, which regulates cellular stress and inflammatory genetic programs through epigenetic mechanisms [126]. Moreover, the SPHK2 selective inhibitor ABC294640 has been reported to have antitumor properties alone or in combination with the multikinase inhibitor sorafenib, an effective treatment in renal cancer, hepatic cancer, and pancreatic adenocarcinoma [63,78,127]. Surprisingly, the sensitivity of PC cells to gemcitabine is enhanced when the ceramide/S1P ratio is increased through the utilization of pharmacologic methods (such as SPHK1 inhibitor or ceramide analog) or small interfering RNA-based approaches that either elevate intracellular ceramide levels or reduce SPHK1 activity [128]. To summarize, exploring the modulation of S1P signaling can offer a promising and innovative approach to treating PC.

#### 4.3.2. Impacting Mitochondria-Mediated Apoptosis of PC Cells

Mitochondria, derived from aerobic bacteria, are pivotal organelles in eukaryotes, and function importantly in the tumorigenesis of PC [129]. Recent studies have indicated that mitochondria may play indispensable roles in the chemoresistance of PC by influencing apoptosis via mitochondrial functions [130,131,132,133,134,135]. It has been found that treatment with ABC294640, an antagonist of SPHKs, significantly delays tumor growth in mice injected with pancreatic ductal adenocarcinoma (PDAC) cells, which is related to the inhibition of apoptosis by the mitochondrial Kv1.3 ion channel blocker PAPTP [136,137,138]. On the other hand, the regulation of S1PRs also modulates mitochondrial-mediated apoptosis in PC cells. SPHK1 antagonist mebendazole was reported to induce apoptosis in PC cells through the intrinsic mitochondrial pathway [139]. Similar conclusions were drawn from the experiments conducted on the PSN1 cells treated with SKI-II [62]. The administration of FTY720 effectively suppresses the growth of PC cells in both humans and mice, resulting in enhanced apoptosis and cell cycle arrest [69]. Furthermore, treatment with either FTY720 or gemcitabine, a drug of choice for PC therapy, whether used alone or in combination, induces mitochondrial depolarization [69].

#### 4.3.3. Inhibiting S1P Signaling Improves PC by Inducing ER Stress of PC Cells

The intersection between ER stress and the ceramide/S1P axis occurs at multiple crucial points in PC. Notably, both anabolic pathway-mediated generation and the S1P lyase-mediated degradation of S1P are processed in ER [140]. Previous research has demonstrated that ER stress activates NF-κB through the activation of receptors located on the plasma membrane: S1PR1, S1PR2, and S1PR3 [141,142]. Conversely, ER stress can stimulate the production of S1P through an increase in SPHK2 expression and utilize it as part of downstream signaling pathways [143]. Evidence from preclinical models of cancer indicates that cancer cells can enhance SPHK2 expression as a survival mechanism in response to ER stress [140]. It has been proven that the dysregulated metabolism of sphingolipids is influenced by both PC and inhibiting S1P can enhance ER stress and immunogenic cell death (ICD), leading to an improved therapeutic response to oxaliplatin in PC. The provided experimental data demonstrating that the induction of ER stress is significantly enhanced by downregulating S1P expression through the knockdown of *Sphk2* or employing the SPHK2 inhibitor ABC294640, at various doses and in different human and murine PC cell lines, resulting in the increased phosphorylation of the PERK/eukaryotic initiation factor 2alpha (eIF2α) pathway and even immunogenic cell death [83]. Moreover, the in vivo findings reveal a notable increase in HMGB1 (a key mediator of ICD) staining intensity, accompanied by the elevated expression of CD3 and CD8 markers in tumors treated with ABC294640 and oxaliplatin, as well as reduced tumor weight [83]. These findings provide compelling evidence for the significance of investigating sphingolipid metabolism in PC and exploring the potential therapeutic targeting of SPHK2/S1P in PC.

#### 4.3.4. Mediating PSCs Activation That Transfers to PC

Tumor–stromal interactions are widely acknowledged to play a pivotal role in the progression of PC [144]. PSCs are stellate-shaped stromal cells situated at the basolateral aspect of acinar cells or in the adjacent perivascular and periductal areas within the normal pancreas [145]. Growth factors, chemokines, cytokines, miRNAs, and exosomes secreted by PSCs have been observed to exert their effects within the tumor microenvironment. They can either activate PSCs through autocrine signaling or elicit paracrine signals on epithelial tumor cells, thereby facilitating the enhanced proliferation, migration, and invasion of these cells [146,147]. The studies of S1P and PSCs are well-documented; it is widely acknowledged that S1P promotes PSC activation in pancreatic-related diseases and primary PSCs [28,59,71]. For instance, the following studies were conducted to provide evidence supporting the role of S1P in triggering PSC activation, leading to the subsequent release of paracrine factors that promote PC cell invasion and growth [28]. They utilized conditioned media obtained from PSCs treated with S1P and applied it to the human pancreatic cancer cell lines, PANC1 and L3.6. The results showed that the conditioned media derived from S1P-stimulated PSCs significantly enhances both the migration and invasion of PANC1 cells, as determined [28]. On the contrary, the inhibition of S1PR2 by JTE-013 or the knockdown of *S1pr2* by *S1pr2*-shRNA resulted in reduced tumor cell migration and invasion compared to control PSCs. Accordingly, in both the orthotopic and subcutaneous models of PC, the growth kinetics of the tumors formed by the co-implantation of *S1pr2*sh PSCs and L3.6 cells were notably slower compared to those formed by the co-implantation of regular PSCs and L3.6 cells, resulting in a significant reduction in the final tumor weight [28].

#### 4.3.5. Being Activated by Bile Acids (BAs)

BAs are widely acknowledged for their capacity to enhance the breakdown and absorption of fats, while also playing a pivotal role in lipid metabolism [148]. Patients diagnosed with PC, particularly those harboring tumors located in the head of the pancreas, frequently encounter biliary obstruction and elevated levels of BAs [149]. In PC cells, BAs diminish the susceptibility to programmed cell death, facilitate progression through the cell cycle, and augment the expression of inflammatory mediators and cellular motility [150]. Furthermore, BAs may disrupt biomembranes at high concentrations [151,152]. Conjugated BAs, such as taurocholic acid (TCA), have been shown to activate S1PR2, and the ERK1/2 and protein kinase B (AKT) signaling pathways [153,154,155]. The growth and migration of S1PR2-expressing cell lines PANC-2-luc and AsPC-1 were enhanced in a dose-dependent manner by the activation of TCA or the S1PR2 agonist CYM5520, while not having any impact on the growth of MIA PaCa-2 and BxPC-3 cells, which primarily exhibit S1PR5 expression. Based on the aforementioned results, an in vivo study was conducted using a murine model of cholestasis associated with metastatic PC, yielding consistent findings with those obtained from the in vitro experiments [58]. The findings suggest that TCA functions as an agonist, activating S1PR2 in pancreatic cells and thereby promoting the development of PC.

Collectively, these studies show that SPHK/S1P signaling promotes cell proliferation, migration, and survival in vitro, as well as enhances tumor growth and activity in vivo. Furthermore, numerous other scholarly articles corroborate this assertion [79,80,156,157,158,159,160]. Yet, the role of S1P in the development and treatment of PC remains a subject of controversy, with some of the literature suggesting its potential beneficial effects while lacking specific mechanistic studies [161,162,163]. Given the above, the present section emphasizes the pivotal role of S1P-dependent mechanisms in tumor growth, proliferation, and metastasis, providing an overview of the current and emerging therapeutic strategies targeting enzymes involved in sphingolipid metabolism and/or signaling for precision PC therapy. 

## 5. S1P Signaling Participates in the Developmental Defects of Pancreas (DDP)

DDP, such as annular pancreas, pancreas divisum, and dorsal pancreatic agenesis, frequently involve congenital genetic abnormalities, which have been identified as a significant etiology for pancreatitis and other gastrointestinal disorders [14,164,165]. The pancreas originates from a small ventral bud and a larger dorsal bud. During the rotation of the foregut, the ventral pancreas undergoes rotational movement towards the dorsal pancreas, ultimately fusing together to form a fully developed pancreas with interconnected ducts [166]. Among the various developmental congenital anomalies, dorsal pancreatic agenesis is one of the rare entities [166]. DDP was reported to be rescued by S1P supplementation [29]. The data suggested that the mechanism of S1P may involve a direct effect on the mesenchyme, which was reported to benefit dorsal pancreatic endoderm [167], instead of a direct effect on the endoderm. The researchers further speculated that blood vessel-derived S1P escaped from the circulation, then bound S1PRs on dorsal pancreatic mesenchymal cells or endothelial cells, stimulating mesenchymal cell proliferation in a cell-autonomous manner or indirectly via intercellular signaling, which rescued dorsal pancreatic growth [29]. Subsequently, the research team conducted further investigations into the impact of S1PR defects on early pancreas development [30]. S1PRs ablation resulted in the reduced volume of the dorsal and ventral pancreata, but it had no effect on endocrine or exocrine differentiation [30]. The defects of the dorsal pancreas in *S1pr*^−/−^ mice were found to be attributed to the reduced proliferation of Pdx1^+^ progenitors, and induced hypervascularization may underlie the adverse impact of S1PR ablation on endodermal growth [30]. Nevertheless, the specific S1PR types and downstream regulatory mechanisms were not clearly clarified in the studies [29,30]. A more recent study confirmed that S1P plays a key role in pancreas development linking specification and lineage allocation through S1PR2 [56]. The findings revealed a delay in pancreas development and severe impairments in both the endocrine and acinar lineages of *S1pr2*-deficient mice embryos. S1P/S1PR2 signaling performed a function through Gαi-mediated yes-associated protein (YAP) stabilization to partially activate connective tissue growth factor (CTGF), as well as attenuate Notch, which participating in regulating the survival of pancreas progenitors [56]. Taken together, these studies highlight the role of S1P signaling in diseases associated with aberrant pancreas development and may propose novel therapeutic strategies.

## 6. Conclusions and Future Prospectives

**Figure 2:**
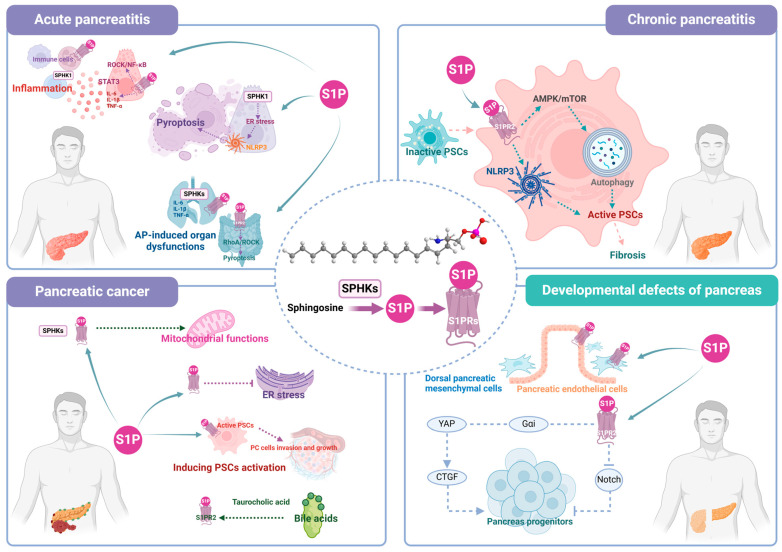
Role and relative mechanisms of S1P signaling in pancreatic diseases.

The fluctuations in the levels of the sphingolipid metabolite S1P, as well as the altered expression of its synthesizing enzymes SPHKs and receptors S1PRs, exert significant impacts on both the physiological and pathological functions of the pancreas. Details are presented in Table 2 and Figure 2. In pancreatitis, S1P signaling exerts local protective effects on the pancreas by modulating inflammatory signals and acinar cell pyroptosis. Moreover, it regulates the extent of pathological injury in AP models through other organs, especially on the lung–intestinal axis. In addition, the underlying mechanism of S1P signaling in CP primarily revolves around the regulation of PSC activation and fibrosis. The regulatory effects of S1P signaling on pancreatitis predominantly involve NF-κB, STAT3 phosphorylation, PERK/TXNIP/NLRP3, RhoA/ROCK, and AMPK/mTOR pathways as well as IFN-γ and TGF-β1. In PC, inhibiting SPHKs/S1PRs can modulate the intrinsic mitochondrial pathway or enhance endoplasmic reticulum stress to impede cancer progression primarily encompassing pathways including PERK/eIF2α, JAK2/STAT3, and FAK/Vimentin, etc. Additionally, the TCA-induced activation of S1PR2 also contributes to PC development. Importantly, targeting the S1P signaling pathway may improve drug sensitivity in PC treatment. Nevertheless, the correlation between S1P signaling and pancreatitis or PC remains controversial. On one hand, it may be crucial for S1P to remain within a certain range, as deviations towards elevated or diminished concentrations can significantly contribute to the progression of these diseases. On the other hand, the role of S1P signaling in these diseases may vary depending on sample types, specific disease models, injury severity levels and time course, as well as variations in the specific receptors it targets. In addition to acquired factors, it is noteworthy that the impact of S1P on pancreatic diseases may also be attributed to congenital developmental abnormalities. Defects in S1P signaling that occur during the embryonic period can lead to the aberrant development of the pancreas, the deficient proliferation of pancreatic progenitors, the loss of endocrine and acinar differentiation, etc. These abnormalities could potentially serve as significant etiological factors underlying pancreatic diseases, such as pancreatitis.

In the future, the potential breakthroughs in S1P signaling in pancreatic diseases warrant further research. The directions can include, but are not limited to the following: (1) Considering the “two-way” effect of S1P signaling in pancreatic diseases, it is necessary to conduct more basic investigations into the role and functional levels of S1P and its specific receptor across diverse cell types and disease contexts. (2) Currently, the majority of studies investigating the mechanism of the S1P receptor in pancreatic diseases primarily focus on S1PR2, with limited research conducted on other receptors. Thus, additional research is warranted to investigate the role of other receptors and explore their in-depth mechanisms. (3) It is of great significance to elucidate whether there are crosstalk or direct correlations between other cell types of neighboring microenvironments, such as nerve cells and immune cells, with pancreatic cells through S1P secretion and associated signaling pathways. (4) There is currently a scarcity of clinical studies exploring S1P signaling as a therapeutic target for pancreatic diseases. Further clinical practice needs to be performed to determine whether S1P signaling can serve as an effective therapeutic intervention. With a more profound comprehension of S1P signaling, future research endeavors focusing on the modulation of S1P signaling, along with the development of SPHK or S1PR agonists/antagonists may emerge as a prominent area of interest, potentially leading to innovative therapeutic interventions for pancreatic diseases.

## Figures and Tables

**Table 2:** Functions of SPHKs/S1P/S1PRs signaling in pancreatic diseases.

Object	Disease	Subject	Model	Treatment	Effects	Mechanism	Ref.
SPHK1	AP	Severe AP patients	/	/	↑Peripheral blood leukocytes SPHK1 in the early stage of severe AP patients.		[95]
SPHK1	AP	Mild and severe AP patients	/	/	↑Peripheral blood leukocytes SPHK1 in the early stage of mild and severe AP patients.	/	[96]
SPHK1/S1PR3	AP	Severe AP patients	/	/	↑SPHK1 and S1PR3 in the early stage of severe AP patients, and then reduced at the restoration stage. SPHK1 expression of peripheral neutrophils, monocytes, and CD4^+^ T lymphocytes was positively correlated with the APACHE II score of severe AP patients.		[97]
S1P	AP	Wistar rats	Cerulein	/	↑Pancreatic S1P and sphingosine; ↓Ceramide	/	[25]
S1P	AP	Severe AP patients	/	/	↓Plasma S1P in severe AP patients.	/	[100]
SPHK2/S1P	AP	AP patients	/	/	↓Serum S1P in AP patients.	/	[101]
		Female C57BL/6 mice	Cerulein	/	↓Serum S1P, pancreatic S1P and SPHK2		
		AR42J cells	Cerulein	/	↓S1P, SPHK2		
S1P	AP	AP patients	/	/	↑Plasma S1P, ↓ceramide in the early stage (days 1 and 3) of mild AP patients, then returned to normal levels at day 7.	/	[102]
					↓Plasma S1P, ↑ceramide in the early stage (days 1 and 3) of severe AP patients, then returned to normal levels at day 7.		
S1PR2	AP	Male ICR mice	Cerulein, injection of TCA into the pancreatic duct	/	↑Pancreatic S1PR2	S1PR2 regulated ROCK/NF-κB signaling	[26]
				Inhibition of S1PR2 by JTE-013, knockdown of *S1pr2*	↓Pancreatic damage, NF-κB.		
		PACs, primary peritoneal macrophages, RAW264.7 cells	TCA	/	↑S1PR2		
				Inhibition of S1PR2 by JTE-013, knockdown of *S1pr2*	↓Cell death, NF-κB, macrophage recruitment and macrophage polarization toward the M1 phenotype.		
S1PR	AP	Female apolipoprotein CIII transgenic C57BL/6J mice	Cerulein	Modulation of S1PR by FTY720	↓Pancreatic pathological injury, MCP-1	/	[107]
S1PR	AP	Female Wistar rats	Injection of 5% sodium taurocholate into the biliopancreatic duct	Modulation of S1PR by FTY720	↓IL-6, IL-10 and TNF-α in plasma/serum, necrosis, inflammation and number of CD4^+^/CD8^+^ cells in pancreas.	/	[66]
S1PR1	AP	Male ICR mice	Cerulein	Activation of S1PR1 by SEW2871	↓Pathological injury of pancreas, serum amylase, lipase, IL-6 and TNF-α, pancreatic MPO, number of CD45^+^CD4^+^ T lymphocytes in the peripheral blood, infiltration of CD4^+^ T cells in pancreas, inflammation.	S1PR1 regulated the phosphorylation of STAT3	[88]
SPHK1	AP	Male C57BL/6J mice	Cerulein	/	↑Pancreatic SPHK1	SPHK1 regulated PERK/TXNIP/NLRP3 signaling	[98]
				Knockout of *Sphk1*	↓Pancreatic damage, pyroptosis, endoplasmic reticulum stress		
		266-6 cells	CCK8	/	↑ SPHK1		
				Knockdown of *Sphk1*	↓LDH, pyroptosis, endoplasmic reticulum stress		
SPHK1	AP	Male SD rats	Injection of 5% sodium taurocholate into the biliopancreatic duct	/	↑SPHK1 in pancreas and peripheral blood neutrophils.	/	[61]
				Inhibition of SPHK1 by SKI 5c	↑Survival rate		
					↓Serum amylase, lipase, TNF-α and IL-1β; MPO in the lung, protein content of bronchoalveolar lavage fluid, pathological injury of the lung.		
S1P/S1PR	AP	Male Wistar rats	Injection of 5% sodium taurocholate into the biliopancreatic duct	100 μg/kg S1P, i.p., once, modulation of S1PR by FTY720	↓IL-1β, IL-6, TNF-α, protein concentration, total cell count, PMN percentage in bronchoalveolar lavage fluid, NF-κB activity of alveolar macrophages, capillary leakage and MPO in the lung. Pathological injury of pancreas and lung.	/	[67]
S1P	AP	Wistar rats	Injection of 5% sodium taurocholate into the biliopancreatic duct	50 μg/kg S1P, i.p., once	↓Protein concentration, leucocyte and neutrophil count of bronchoalveolar lavage fluid, MPO of the lung tissue, pathological injury of the lung.	/	[114]
S1PR2	AP	Male C57BL/6 mice	Cerulein + lipopolysaccharide	Inhibition of S1PR2 by JTE-013	↓Pathological injury of pancreas and intestine, inflammation, intestinal tissue injury and pyroptosis.	S1PR2 regulated RhoA/ROCK signaling	[57]
		THP-1 cells	Lipopolysaccharide +ATP	Knockdown or overexpression of *S1pr2*	S1PR2 positively regulated macrophage pyroptosis, and negatively regulated cohesin expression in FHC cells after co-culture of FHC and THP-1 cells.		
S1P/S1PR2	CP	Male Wistar rats	Dibutyltin dichloride	/	↑Plasma and pancreatic S1P	S1P binding to S1PR2 promoted PSC activation and pancreatic fibrosis in CP by regulating autophagy and the NLRP3 inflammasome	[71]
				200 μg/kg/day S1P, i.p., 4 weeks	↑Pancreatic damage, fibrosis, autophagy, S1PR2, NLRP3		
		PSCs	/	5 μM S1P, 24h	↑PSC activation, autophagy, S1PR2, NLRP3		
				Inhibition of S1PR2 by JTE-013, knockdown of *S1pr2*	↓PSC activation, autophagy, NLRP3		
SPHK1/S1P/S1PR2	CP	C57BL/6 mice	Cerulein, pancreatic duct ligation	/	↑Serum S1P, pancreatic SPHK1, S1PR2	PACs-derived S1P contributed to fibrosis of CP via inducing autophagy and activation of PSCs through the AMPK/mTOR signaling	[59]
				Knockout of *Sphk1*, inhibition of SPHK1 or S1PR2 by PF-543 and JTE-013, respectively	↓Pathological injury of pancreas, fibrosis, inflammation, atrophy of the pancreas.		
		PACs	CCK, hypoxia	/	↑SPHK1, S1P in PACs		
				Knockout of *Sphk1* in PACs, PSCs treated with S1P, knockdown of *S1pr2* or inhibition of S1PR2 in PSCs	SPHK1/S1P/S1PR2 signaling positively regulated activation and autophagy of PSCs.		
S1PR	CP	Male WBN/Kob rats	/	Modulation of S1PR by FTY720	↑Pancreas weights	S1PR regulated IFN-γ and TGF-β1 expression	[68]
					↓Pancreatic MPO activity, hydroxyproline content, pathological injury of pancreas, inflammation, fibrosis, necrosis, infiltration of CD4- and CD8-positive T cells in the pancreas.		
S1P	PC	C57BL/6 mice	PANC-2 cells, subcutaneous	PhotoImmunoNanoTherapy	↓Tumor volume	/	[126]
		Athymic nude mice	BxPC-3-GFP cells, orthotopic	PhotoImmunoNanoTherapy	↑Surem S1P		
SPHK2	PC	BxPC-3 cells	/	Inhibition of SPHK2 by ABC294640, sorafenib	↓Cell viability, ↑cell apoptosis	/	[78]
		SCID mice	BxPC-3 cells, subcutaneous	Inhibition of SPHK2 by ABC294640, sorafenib	↓Tumor growth, ↑Tumor cell apoptosis		
SPHK2	PC	SCID mice	BxPC-3 cells, subcutaneous	Inhibition of SPHK2 by ABC294640	↓Tumor growth	/	[63]
SPHK1	PC	BxPC-3 or PANC-1 cells	Gemcitabine	Inhibition of SPHK1 by SKI or knockdown of *Sphk1*	↓Cell viability	/	[128]
				Overexpression of *Sphk1*	↑Cell viability		
SPHKs/S1P	PC	C57BL/6 mice	PDAC cells, metastatic	PAPTP + ABC294640	↓Tumor growth	SPHKs regulated the mitochondrial Kv1.3 ion	[138]
		MIA PaCa-2 cells	Inhibition of Kv1.3 by PAPTP	/	↑Sphingosine, S1P-phosphatase		
				Inhibition of S1P-phosphatase by XY-14	↓Sphingosine, death of pancreas cancer cells		
				Inhibition of SPHK2 by ABC294640	↑Sphingosine, death of pancreas cancer cells		
SPHK1/S1P	PC	MIA PaCa-2, PANC-1 or Capan-1 cells	/	Inhibition of SPHK1 by mebendazole	↓Cell migration, proliferation and viability, ↑cell mitochondrial apoptosis	SPHK1 regulated the intrinsic mitochondrial pathway, JAK2/STAT3 and FAK/Vimentin signaling pathway	[139]
SPHKs	PC	PSN1 cells	/	Inhibition of SPHKs by SKI-II	↓Cell proliferation	SPHKs regulated the ratio of S1P/C16 Cer	[62]
S1PR1	PC	MIA PaCa-2 or PAN02 cells	/	Inhibition of S1PR1 by FTY720	↓Cell migration, proliferation	S1PR1 regulated the mitochondrial membrane potential, S1PR1-STAT3 loop, and epithelial to mesenchymal transition	[69]
		MIA PaCa-2 or PAN02 cells	Gemcitabine	Inhibition of S1PR1 by FTY720	↑Cell death, ↓cell proliferation		
		NOD.CB17-Prkdcscid/J mice or C57BL/6 mice	Luciferase-tagged MIA PaCa-2 cells	Treatment of FTY720 and gemcitabine	↓Tumor volume, tumor cell metastasis, proliferation, ↑Cell apoptosis or necrosis		
SPHK2	PC	MIA PaCa-2 or PANC-1 cells	Oxaliplatin	Inhibition of SPHK2 by ABC294640 or knockdown of *Sphk2*	↓Cell viability, ↑ER stress	SPHK2 modulated ER stress, thereby regulating PERK/eIF2α phosphorylation and ICD	[83]
		Male C57BL/6 mice	KPC cells, orthotopic	Oxaliplatin +ABC294640	↓Tumor weight, ↑ICD		
S1P/S1PR2	PC	PANC-1 or L3.6 cells	Conditioned media collected from S1P-treated PSCs	/	↑Cell proliferation, migration	S1P regulated tumor microenvironment and the interactions of PSCs with cancer cells	[28]
		PANC-1 cells	Conditioned media collected from S1P-treated PSCs	Inhibition of S1PR2 by JTE-013, Knockdown of *S1pr2*	↓Cell migration, invasion		
		Male nude mice	L3.6 cells + PSCs or ASPC-1 cells + PSCs, orthotopic	/	↑Tumor volume, weight, metastasis		
				Knockdown of *S1pr2*	↓Tumor volume, weight, metastasis		
S1PR2	PC	PANC-1 or CFPAC-1 cells	Gemcitabine	Inhibition of S1PR2 by JTE-013	↑Gemcitabine-induced apoptosis, ↓Cell migration, invasion	TCA contributes to gemcitabine ineffectiveness by activating S1PR2/ERK signaling	[155]
S1P/S1PR2	PC	Male C57BL/6 mice	PANC-2-luc cells + bile duct ligation, orthotopic, metastatic	Activation of S1PR2 by CYM5520	↑Tumor growth	/	[58]
				Treated with anti-S1P-antibody, sphingomab	↓Survival, ↑tumor burden		
		PANC-2-luc or ASPC-1 cells	TCA	/	↑Cell growth		
				Inhibition of S1PR2 by JTE-013	↓Cell growth, migration, viability		
				Inhibition of all S1PRs except S1PR2 by FTY720	↑Cell viability		
SPHKs	PC	PAN02 cells	/	Knockdown of *Sphk1*	↑Cell proliferation, migration	/	[27]
		PAN02 cells	/	Knockdown of *Sphk2*	↓Cell proliferation, migration		
		Male C57BL/6 mice	*Sphk1* KO PAN02 cells	/	↑Survival		
		Male C57BL/6 mice	*Sphk2* KO PAN02 cells	/	↓Survival		
Sphingosine	PC	Male nude mice	PANC-1 or PANC-1 TRCs, orthotopic	/	Sphingosine significantly decreased in TRCs	/	[156]
SPHK2	PC	Male C57BL/6 mice	PAN02 cells	Inhibition of SPHK2 by ABC294640	↓Tumor growth	/	[157]
S1P	PC	Capan-1 or PANC-1 cells	/	S1P, 0.5 and 1 μΜ; Inhibition Src by PP2	↓Cell proliferation, migration	/	[158]
SPHK1	PC	BxPC-3 cells	/	Overexpression of *Sphk1*	↑Cell proliferation, migration	/	[159]
SPHK1	PC	SWl990 cells	/	Inhibition of SPHK1 by DMS	↓Cell proliferation, ↑cell apoptosis	/	[80]
				Activation of SPHK1 by phorbol 12-myristate13-acetate	↑Cell proliferation, ↓cell apoptosis		
S1P/S1PRs	PC	PANC-1 cells	/	S1P, 20–200 nΜ; Inhibition of S1PRs by VPC23019	↓Cell migration, invasion	/	[79]
S1P	PC	Female athymic nude mice	PANC-2-SAL, TPAN1-IFA, metastatic	Inhibition of S1P Lyase by LX2931	↓Tumor volume, hypoxia marker	S1P regulated tumor hypoxia and therapy efficacy in solid tumors	[161]
S1PR2	PC	Nude mice	HPAF II tumor cells expressing S1PR2-GFP	/	↓Tumor size and metastatic frequency	/	[162]
S1P	PC	PANC-1 or MIA PaCa-2 cells	/	S1P, 0–10 μΜ	↓DNA synthesis	/	[163]
SPHK1	PC	C57BL/6 mice	PANC-2-luc cells	Knockout of *Sphk1*	↓Tumor burden, cell proliferation	/	[160]
S1P	DDP	In vitro culture of pancreatic explants of mice embryos	N-cadherin knockout	S1P, 0.1 µM	↑Early morphogenesis of the dorsal pancreas, formation of the dorsal pancreatic bud, dorsal pancreatic mesenchymal cell proliferation, development of dorsal pancreatic endoderm, mesenchyme and endothelium	S1P stimulated mesenchymal cell proliferation	[29]
S1PR	DDP	C57BL6 mice embryos	/	Knockout of *S1pr*	↓Volume of the dorsal and ventral pancreata, proliferation of Pdx1^+^ progenitors	S1PR regulated proliferation rate of Pdx1^+^ progenitors, hypervascularization	[30]
					↑Aberrant development of the pancreatic endoderm, vascular density of dorsal pancreas		
S1PR2	DDP	C57BL/6J mice embryos	/	Knockout of *S1pr2*	↓Development of pancreas, survival and commitment of pancreas progenitors, endocrine and acinar differentiation	S1PR2 regulated Gαi-mediated YAP stabilization to partially activated CTGF, and attenuated Notch	[56]

“↑” means up-regulation; “↓” means down-regulation.

## Abbreviations

ABC: ATP-binding cassette; AKT, protein kinase B; AMPK, adenosine monophosphate-activated protein kinase; AP, acute pancreatitis; ATP, adenosine triphosphate; BAs, bile acids; CP, chronic pancreatitis; CTGF, connective tissue growth factor; DDP, developmental defects of pancreas; eIF2α, eukaryotic initiation factor 2alpha; ER, endoplasmic reticulum; ERK1/2, extracellular signal-regulated kinase 1/2; FAK, focal adhesion kinase; JAK2, Janus kinases2; ICD, immunogenic cell death; LPPs, lipid phosphate phosphohydrolases; MCP-1, monocyte chemotactic protein-1; mTOR, mammalian target of rapamycin; NF-κB, nuclear factor-kappa B; NLRP3, Nod-like receptor family, pyrin domain containing 3; PACs, pancreatic acinar cells; PC, pancreatic cancer; PDAC, pancreatic ductal adenocarcinoma; PERK, protein kinase RNA-like endoplasmic reticulum kinase; PI3K, phosphatidylinositide 3-kinase; PSCs, pancreatic stellate cells; RhoA, Ras homolog family member A; ROCK, Rho-associated kinase; S1P, sphingosine-1-phosphate; S1PL, S1P lyase; S1PRs, S1P receptors; SPHKs, sphingosine kinases; SPNS2, spinster homolog 2; SPPs, S1P phosphatases; STAT3, signal transducer and activator of transcription 3; TCA, taurocholic acid; TGF, transforming growth factor; TNF, tumor necrosis factor; TXNIP, thioredoxin-interacting protein; YAP, yes-associated protein.
